# Alterations in the ribosomal machinery in cancer and hematologic disorders

**DOI:** 10.1186/1756-8722-5-32

**Published:** 2012-06-18

**Authors:** Niraj Shenoy, Rachel Kessel, Tushar D Bhagat, Sanchari Bhattacharyya, Yiting Yu, Christine Mcmahon, Amit Verma

**Affiliations:** 1 Albert Einstein College of Medicine, 1300 Morris Park Avenue, Bronx, NY, 10467, USA

**Keywords:** Ribosome, MDS, Anemia

## Abstract

Ribosomes are essential components of the protein translation machinery and are composed of more than 80 unique large and small ribosomal proteins. Recent studies show that in addition to their roles in protein translation, ribosomal proteins are also involved in extra-ribosomal functions of DNA repair, apoptosis and cellular homeostasis. Consequently, alterations in the synthesis or functioning of ribosomal proteins can lead to various hematologic disorders. These include congenital anemias such as Diamond Blackfan anemia and Shwachman Diamond syndrome; both of which are associated with mutations in various ribosomal genes. Acquired uniallelic deletion of RPS14 gene has also been shown to lead to the 5q syndrome, a distinct subset of MDS associated with macrocytic anemia. Recent evidence shows that specific ribosomal proteins are overexpressed in liver, colon, prostate and other tumors. Ribosomal protein overexpression can promote tumorigenesis by interactions with the p53 tumor suppressor pathway and also by direct effects on various oncogenes. These data point to a broad role of ribosome protein alterations in hematologic and oncologic diseases.

## Introduction

Eukaryotes have 80 S ribosomes, each consisting of a small (40 S) and large (60 S) subunit. The large subunit is composed of a 5 S RNA (120 nucleotides), a 28 S RNA (4700 nucleotides), a 5.8 S subunit (160 nucleotides) and approximately 49 proteins. The 40 S subunit has a 18 S RNA (1900 nucleotide) and approximately 33 proteins.

The biogenesis of the ribosome machinery is a highly coordinated process. It is composed of the synthesis and processing of ribosomal RNA (28SRNA, 18SRNA and 5.8SRNA in the nucleolus and 5SRNA in the nucleoplasm), synthesis of ribosomal proteins in the cytoplasm and their import into the nucleus, assembly of rRNA and ribosomal proteins in the nucleoplasm and finally transport of the mature subunits (40 S and 60 S) into the cytoplasm.

The basic translation machinery is composed of ribosomal subunits, mRNAs, transfer RNAs (tRNAs), and translational initiation and elongation factors. First, the initiation factors eIF2, eIF3, tRNA and GTP are incorporated into a 40 S ribosomal subunit to form a 43 S complex. Second, eIF4E is recruited into the 43 S complex to form a 48 S complex with mRNA. Finally, a 60 S ribosomal subunit and the 48 S subunit form the final 80 S complex [1].

## Both ribosomal proteins and ribosomal RNA have important functional roles

The relative functional significance of the rRNAs and the ribosomal proteins has been a matter of interest for many years. In the 1970s, it was generally accepted that ribosomal proteins constituted the functionally active part of the ribosome, whereas rRNA was essentially a scaffold that kept the proteins in position for optimal functioning. By the 1980s, the pendulum had swung, with ribosomal proteins thought to be the scaffolding for the rRNA. The reason for this change was the discovery of catalytic RNA and data showing the direct involvement of rRNAs in distinct ribosomal functions, for example, the Shine-Dalgarno interactions between mRNA and rRNA during initiation of bacterial protein synthesis, as well as the predominance of rRNA at the decoding and peptidyl-transferase centre (PTC) of the ribosome. The crystal structures of the ribosome confirmed this predominance of rRNA at these active sites, but also revealed that a number of ribosomal proteins were located in positions of functional importance, for example, RPS12 at the decoding center, RPL11 and RPL10 as components involved in translation factor binding. Over the years, specific functions of many individual ribosomal proteins were identified and currently it is accepted that both proteins and RNA are essential for the optimal functioning of the ribosome.(Wilson, D. N. and Nierhaus, K. H. 2006).

Futhermore, several ribosomal proteins have been found to have extraribosomal functions including DNA repair; autoregulation of ribosomal protein synthesis and translation and regulation of development of malignant transformation mainly through interaction with p53. In E.coli, in addition to these functions, ribosomal proteins have been found to play a role in DNA replication, transcription and RNA processing [2,3]. The involvement of these extraribosomal functions in the pathogenesis of ribosomopathies and cancer is reviewed in this article.

## Mutations or deletions of ribosomal proteins can result in ribosomopathies (summarized in Table 1)

**Table 1:** Salient features of various ribosomopathies

**SYNDROME**	**GENE INVOLVED**	**CLINICAL FEATURES**	**LABORATORY FEATURES**
DIAMOND BLACK- FAN ANEMIA	RPS 19 (25%); RPS 24; RPS 17; RPL35A; RPL5; RPL11; RPS 10; RPS 26	Craniofacial defects; pallor; short stature; thumb abnormalities	Macrocytic anemia; elevated HbF levels; elevated ADA; parvovirus B19 seropositivity (50% by age 15)
SHWACHMAN DIAMOND SYNDROME	SBDS 7q11. 21 (90%)	Recurrent infections; pancreatic insufficiency; short stature; increased risk of malignancy (AML, MDS); increased risk of CVID	Neutropenia; thrombocytopenia; aplastic anemia; neutrophil chemotaxis defect; low immunoglobulins; low B cells
CARTILAGE HAIR HYPOPLASIA	RMRP (RNase Mitochondroal RNA processing) gene	Short limbed dwarfism; metaphyseal chondrodysplasia; hypoplastic hair; recurrent infectiions (Pneumocystis, CMV)	Neutropenia; lymphopenia; hypogammaglobulinemia; defective B cells; reduced T cell count
TREACHER COLLINS SYNDROME	TREACLE GENE (TCOF1 mutations)	Craniofacial abnormalities (micrognathia, ear deformities, macrostomia, hearing loss, anti-mongoloid slant)	Anemia; T cell abnormalities; hypogammaglobulinemia
DYSKERATOSIS CONGENITA	DKC1 at chr.X q28	Nail dystrophy; reticulated skin pigmentation; oral leukoplakia; infections; liver, lung fibrosis	Reduced telomere length; low IgM and B cell count
5q- SYNDROME	RPS14	Pallor; progression to AML (10%)	Macrocytic anemia
TURNER SYNDROME	HAPLOINSUFFICIENCY OF RPS4X HYPOTHESIS	Short stature; webbed neck; gonadal dysgenesis; mental retardation; CVS malformations	45XO karyotype; hypogammaglobulinemia; low T cell count; CVID

RPS: Ribosomal protein of small subunit; RPL: Ribosomal protein of large subunit; SBDS: Shwachman-Bodian-Diamond syndrome; RMRP: RNase Mitochondroal RNA processing; DKC: Dyskeratosis congenital; CVID: Common Variable Immunodeficiency.

Ribosomopathies are disorders resulting from impaired ribosome biogenesis and function. Diamond-Blackfan anemia and 5q- syndrome are the two clinical syndromes for which there is abundant genetic and experimental evidence that the impairment in erythropoiesis is due to mutations in ribosomal genes. However, there are a few other clinical syndromes in which ribosome dysfunction is thought to play a role and recently, ribosomal dysfunctions have been identified in some malignancies as well.

1. Diamond-blackfan anemia (DBA):DBA is a congenital aregenerative anemia accompanied by erythroblastopenia (less than 5% of nucleated cells in the bone marrow are erythroblasts). It is often discovered before the age of two years. There are associated developmental malformations in 40% of the cases. The malformations most often involve the head and neck (palatine cleft, ogival palate, webbed appearance of the neck, microphthalmia, absent lower eyelashes, deformed external ears, micrognathia), limbs (triphalangeal thumb, supernumerary digits, syndactyly), urogenital tract, heart and/or the spinal cord.In 1999, Draptchinskaia reported mutations in the gene encoding ribosomal protein S19 in patients with Diamond-Blackfan anemia [4]. Since then, mutations in a number of ribosomal proteins have been identified in 50% of DBA patients. Mutations in RPS19 and RPS 24, which were the first two mutations identified, were shown to impair the pre-RNA processing of 18 S RNA which in turn lead to reduced production of 40 S ribosomal subunit and mature 80 S ribosomes [5-7]. The other ribosomal protein gene mutations found in DBA involve RPS7, RPS15, RPS17, RPS27A, RPL5, RPL11, RPL35A and RPL36 [8-10]. Current therapy for DBA includes steroids and chronic blood transfusions, with the only definitive therapy being bone marrow transplantation [11].

2. 5q- syndrome:5q- syndrome is a disorder resulting from deletion of the long arm of chromosome 5 and is characterized by macrocytic anemia with normal/elevated platelets with hypolobulated micromegakaryocytes. It is found predominantly in females of advanced age. It is now classified as an independent subtype of MDS, with better prognosis and a relatively low rate (10%) of progression to acute myeloid leukemia compared with other subtypes of MDS [12]. In patients with 5q- syndrome, 1 allele of RPS14 is deleted and haploinsufficient expression of RPS14 has been found in patients [5,13,14]. Decreased expression of RPS14 causes ineffective erythropoiesis. It has also been shown that reexpression of RPS14 in samples from patients with 5q- syndrome rescues erythropoiesis. As discussed earlier, RPS14 deficiency causes impaired processing of 18 S RNA and in turn leads to reduced levels of the 40 S subunit [15]. The heterozygous deletions of chromosome 5q are large and haploinsufficiency of other genes may also contribute to the 5q- phenotype [16]. For example, haploinsufficiency of micrornas, miR-145 and miR-146a, may be responsible for thrombocytosis seen in this disorder [17,18]. However, the erythroid defect, which is the aspect of the phenotype most analogous to DBA, has been shown both in in-vivo (murine) and in-vitro models to be a result of haploinsufficiency of RPS14 [15,19]. Lenalidomide has been shown to be very effective in the treatment of patients with 5q- syndrome. In a phase 2 trial in low risk MDS patients with 5q deletions, lenalidomide decreased transfusion requirements in 76% and 61% had a complete cytogenetic response [20,21].

3. Treacher collins syndrome (TCS):Also called mandibulofacial dysostosis, TCS is a rare autosomal dominant congenital disorder characterized by craniofacial abnormalities such as micrognathia, malformed or absent external ears, underdeveloped zygoma, downward slanting eyes, conductive hearing loss, colobomata of the lower eye lids, cleft palate, brachycephaly, variable degree of facial nerve involvement [22]. Intelligence of patients with TCS is usually normal but psychosocial problems associated with the facial deformity affect the quality of life of a number of patients. In 1996, TCOF1 gene mutation was found to be the cause for TCS. TCOF1 encodes a protein called Treacle, which is a constituent of one of the preribosomal ribonucleoprotein complexes [23]. It has been shown to be essential for the transcription of ribosomal DNA and it may also be involved in the methylation of rRNA [24]. Mice haploinsufficient for TCOF1 have diminished production of ribosomes and this has been shown to correlate with decreased production of neural crest precursor cells [25]. This in turn leads to decreased number of neural crest cells migrating to the first and second pharyngeal arches, resulting in the development of the syndrome. An interesting study by Jones et al. showed that chemical and genetic inhibition of p53 in these mice could prevent the development of craniofacial abnormalities [26]. The link between ribosomopathies and p53 activation will be discussed later in the review.

4. Cartilage hair hypoplasia:CHH is an autosomal recessive disorder that results in short limb dwarfism [27]. It is associated with T cell and B cell immunodeficiency. Hematologic abnormalities can also include macrocytic anemia, apart from lymphopenia. Management is supportive, with stem cell transplantation being the only curative option. It is indicated specially in patients with severe T cell deficiency, although transplantation does not cure skeletal abnormalities [28]. The genetic defect in cartilage hair hypoplasia has been identified as a mutation in the gene for RMRP, mapped to 9p12 [29]. RMRP is a ribonucleoprotein present in the nucleus and mitochondria. RMRP is involved in the cleavage of RNA in mitochondrial DNA synthesis and cleavage of pre-ribosomal RNA (pre-RNA) [30]. RMRP is required for cell growth, consistent with observations that a generalized defect in cell growth is seen in T cells, B cells and fibroblasts. In 2005, a study reported that CHH cells with RMRP mutation were found to have increased levels of cyclin B2 mRNA [31]. Cyclin B2 is known to contribute to chromosomal instability through alterations of the mitotic spindle checkpoint, which suggests another explanation for the bone marrow dysfunction [32].

5. Shwachman diamond syndrome (SDS):SDS is a rare autosomal recessive disorder characterized by exocrine pancreatic insufficiency, bone marrow dysfunction, skeletal abnormalities and a predisposition to leukemia [33]. It is the third most common inherited bone marrow failure syndrome after Fanconi anemia and Diamond Blackfan anemia. Patients typically present in early infancy with malabsorption, steatorrhea, failure to thrive and deficiency of Vitamins A, D, E and K. Neutropenia is the most common hematologic abnormality seen in patients. Data from a large international cohort study consisting of 88 patients showed neutropenia in 98% of the patients, anemia in 42%, thrombocytopenia in 34% and pancytopenia in 19%. In 2003, Boocock et al. reported causal mutations in the SBDS gene, named after Shwachman-Bodian-Diamond, who first reported the syndrome. About 90% of the patients were found to have biallelic mutations of the SBDS gene [34]. Although the precise function of the gene is unclear, there is evidence suggesting a role in ribosome biogenesis and ribosome processing [35]. SDS cells have been shown to underexpress many ribosomal protein genes including RPS9, RPS20, RPL6, RPL15, RPL22, RPL23 and RPL29 and genes involved in rRNA and mRNA processing [36]. SBDS functions in ribosome synthesis by promoting the recycling of eukaryotic initiation factor 6 (eIF6) in a GTP-dependent manner. Finch et al. stated that a perturbation in this function causes SBDS [37]. However, SBDS is a multifunctional protein and it is also possible that non ribosomal mechanisms have a dominant role in the pathogenesis of the syndrome. Of note is SBDS’ role in stabilizing the mitotic spindle [38], the absence of which may lead to chromosomal instability and therefore bone marrow failure.

6. Dyskeratosis congenita (DKC):DKC is a rare, progressive bone marrow failure syndrome characterized by the triad of reticulated skin hyperpigmentation, nail dystrophy and oral leukoplakia. Early mortality is often associated with bone marrow failure, infections, fatal pulmonary complications or malignancy [39]. In all cases of DKC, the causative mutations are in the telomerase complex, composed of telomerase reverse transcriptase (TERT), telomerase RNA (TERC) and dyskerin, which adds specific DNA sequences to the ends of chromosomes and counters some of the normal shortening that occurs during DNA replication [40]. In X-linked recessive DKC, which has a more severe phenotype compared to the autosomal dominant DKC, the mutation occurs in the DKC1 gene, which codes for dyskerin [41]. Apart from being a part of the telomerase complex, dyskerin is also involved in rRNA modification (pseudo-uridylation of rRNA). However, the functional consequence of the defect in pseudouridylation of rRNA remains unclear. Patients with the autosomal dominant form of DKC have mutations in TERC; and two families with the autosomal recessive form of DKC have mutations in TERT which suggest that a defect in telomerase activity alone is sufficient for defective hematopoiesis [42]. However, a study on a DKC1 mutant mouse showed pseudouridylation of rRNA before the manifestation of clinical features of DKC whereas the reduction in telomere length became evident only in the later generations [43]. This suggests that in addition to the telomerase complex mutations, defects in rRNA modification might have a contributory role in the clinical manifestations of DKC.

7. Common variable immunodeficiency (CVID):CVID is a disorder that involves low levels of most or all of the immunoglobulin classes, a lack of B lymphocytes or plasma cells that are capable of producing antibodies and frequent bacterial infections. A diagnosis of CVID is reserved for those with an undefined B cell dysfunction. About 50% of the patients also have T lymphocyte dysfunction. The recognition of a patient with DBA who subsequently developed CVID and the finding of a mutation in the SBDS gene in another patient with CVID has led to the hypothesis that ribosome biogenesis defects are also responsible for a subset of CVID. A review of the literature shows that patients with ribosomal defects may share abnormalities of T and B cell development with many features of CVID, and which may not be recognized as such by non-immunologists [44].

8. Turner syndrome:Turner syndrome results from a chromosomal abnormality in which all or part of one of the female sex chromosomes is absent. Monosomy X in which an entire X chromosome is missing, is most common. Characteristic physical abnormalities include short stature, webbed neck, broad chest, low hairline, low-set ears and edema. Girls with Turner Syndrome typically experience gonadal dysfunction resulting in amenorrhea and sterility. Other health problems frequently present include congenital heart disease, hypothyroidism, diabetes, vision and hearing problems, autoimmune diseases and developmental delays.Human sex linked genes RPS4X and RPS4Y encode 2 isoforms of ribosomal protein S4. RPS4 is a component of 40 S subunit and haploinsufficiency of RPS4 is hypothesized to lead to some features of Turner’s syndrome such as panhypogammaglobulinemia, low IgM, decreased T and B cell numbers [45,46].

### Ribosomal protein disorders associated with malignancies

1. HEPATOCELLULAR CANCER: Increased expression of ribosomal protein S2 was found in mouse hepatocellular carcinoma (HCC) samples and in mouse livers after partial hepatectomy. RPS2 is involved in amino-acyl tRNA binding to ribosome, thus potentially affecting the fidelity of mRNA translation. Increased levels of RPS2 were shown to correlate with increased cell proliferation. However, whether the overexpression of RPS2 is a causative factor or just an associated phenomena in increased cell proliferation is unclear [47]. Similarly, enhanced expression of S8, L12, L23a, L27 and L30 ribosomal protein mRNAs were found in three human HCC cell lines (Huh-7, Hep G2 and HLF) [48]. Recently, RPL36 was found to be expressed in 45 of 60 HCC patients by immunohistochemistry. Expression of RPL36 was found to be higher in the early tumor stages (I, II). Patients with RPL36 expression revealed better overall survival (p = 0.037) and by multivariate survival analysis, it was found to be an independent prognostic factor for overall survival (p = 0.026) in resected HCC. Thus, RPL36 may be involved in the early stage of hepatocarcinogenesis and could be used as a potential prognostic marker in patients with resected HCC [49].

2. Colorectal cancer: Increased expression of ribosomal protein genes, including S3, S6, S8, S12, L5 has been reported in colorectal cancer (CRC) [50]. Another study demonstrated that 12 ribosomal proteins (RPSa,S8,S11,S12,S18,S24,L7, L13a,L18,L28,L32,L35a) were differentially expressed in CRC when compared to healthy colonic mucusa. Immunohistochemical examination of 18 CRC and paired normal mucosa samples showed that RPS11 and RPL7 were highly expressed in CRC (especially in immature mucosal cells located in the crypt base) but could also be detected at lower levels of expression in the normal mucosa [51]. The mRNA levels for some specific ribosomal proteins were associated with the Dukes’ stage of the tumor [52]. Most of the studies have been descriptive studies that do not implicate these proteins in carcinogenesis in any functional analysis.

3. Prostate cancer : The RPL19 gene has been found to be highly overexpressed in prostate cancer cell line. An inverse relationship between RPL19 expression and patient survival has been found, with patients expressing low levels of RPL19 surviving significantly longer. The statistical significance was also comparable to the Gleason score (p < 0.05) suggesting that RPL19 could be used as an independent prognostic marker [53]. Furthermore, siRNA knockdown of RPL19 gene abrogated the aggressive phenotype of human prostate cancer [54]. Recently, the RPS2 protein was reported to be a novel therapeutic target in prostate cancer. A 'ribozyme-like' DNAZYM-1P '10-23' motif oligonucleotide was developed to knock down RPS2 expression in malignant cells and results showed that DNAZYM-1P inhibited cell growth and induced apoptosis in malignant prostate cell lines and had little effect on the normal cell lines. It was the first time that therapeutic targeting of a ribosomal protein was shown to have excellent results in pre-clinical tumour modelling studies [55].

4. Malignant melanoma : A single chain ribosome inactivating protein (scRIP), developed from the cytotoxic A subunit of Shiga- like Toxin 1 (SLT-1A), has been shown to have the ability to kill 7 of 8 melanoma cell lines. The protein called SLT-1A^IYSNKLM^ harbors the 7 amino acid peptide insertion IYSNKLM, allowing it to selectively attack the melanoma cells and spare normal cells. In the same study it was shown that mice with the human melanoma xenograft had better survival and tumor regression with a combination of SLT-1A^IYSNKLM^ and Dacarbazine than either alone [56].

5. Metastatic sarcoma : The serine/threonine kinase, mammalian target of rapamycin (mTOR), is a protein kinase of the phosphatidylinositol 3-kinase (PI3K)/AKT signaling pathway thought to have a key role in controlling cancer growth and thus is an important target for cancer therapy. Several inhibitors of mTOR are in clinical trials, including AP23573, which is being tested on metastatic sarcomas and other tumors. Phosphorylated S6 ribosomal protein is a marker for the activity of mTOR and it was found that the level of phosphorylated S6 ribosomal protein expression was predictive of early tumor response to the mTOR inhibitor. Patients with a high expression of phosphorylated S6 responded better to the mTOR inhibitors, suggesting that this is a promising predictive sarcoma marker for targeted mTOR inhibitor therapy [57].

6. Non hodgkins lymphoma: RPS6 is highly expressed in Diffuse large B cell lymphomas and genetic modulation of RPS6 protein levels with specifically targeted short hairpin RNAs (shRNA) lead to a decrease in the actively proliferating population of cells compared with control shRNA. RPS6 was shown to associate with multiple mRNAs containing a 5’TOP tract, which encode the translation machinery [58].

## Functional relevance of ribosomal proteins in tumorigensis

**Figure 1:**
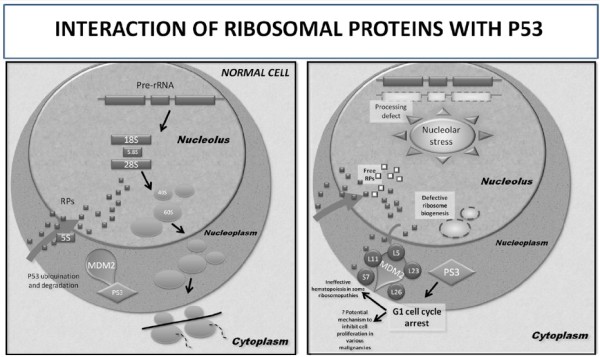
**Loss of ribosomal proteins activats p53:** In a normal cell, MDM2 binds to p53 in the nucleoplasm, leading to p53 ubiquination and degradation. Defective ribosome biogenesis leads free ribosomal proteins moving out of the nucleolus and binding MDM2, thereby preventing the degradation of p53 which then causes G1 cell cycle arrest.

1. Protein biosynthesis function of ribosomal proteins in cancer: Both tumor suppressors and oncogenes have been shown to modulate the ribosome protein biosynthesis as well as ribosome translation initiation in various models [59].. MYC, a proto-oncogene product, regulates the mature ribosome biogenesis by modifying the genes of necessary factors involved in ribosomal assembly. Its overexpression in tumor cells increases the expression and activity of ribosomal components. Therefore, regulation of protein synthesis could be an important mechanism by which MYC regulates cell growth and initiates tumorigenesis [60]. PTEN, a tumor suppressor, also regulates the mature ribosome formation, by suppressing the activity of RPS6K [61]. These data suggest that perturbation in the protein biosynthesis fuction of ribosomal proteins may be an important contributor to carcinogenesis.

2. Interaction of p53 with ribosomal proteins: Likely mechanism involved in some features of ribosomopathies and associations between ribosomal proteins and cancer.A number of events, including haploinsufficiency of some ribosomal proteins, impart instability to ribosomal biogenesis and cause nucleolar stress. In response to this stress, some ribosomal proteins (RPL5, RPL11, RPL23, RPS7 and RPL26) bind to MDM2 and block MDM2-mediated p53 ubiquitination and degradation, resulting in p53-dependent cell cycle arrest. By doing so, the ribosomal proteins play a crucial role in connecting deregulated cell growth with inhibition of cell division. The ribosomal protein-MDM2-p53 signalling pathway provides a molecular switch that may constitute a surveillance network monitoring the integrity of ribosomal biogenesis [62]. (Figure 1) In the *Rps19* mutant mouse model used to study DBA, induction of p53 and p53 target genes was identified with the mutation. These mice had decreased hematocrit and increased MCV and phenocopied the human disease. The anemia observed in these mice was partially abrogated with monoallelic inactivation of p53. Homozygous inactivation of p53 in *Rps19* mutant mice fully corrected the hematologic abnormality [63]. Furthermore, when the mice with the conditional deletion of a set of genes found in the common deleted region of the 5q- syndrome were crossed with p53 null mice, there was a complete rescue of the erythroid phenotype [19]. These findings indicate that p53 induction is critical for the macrocytic anemia caused by ribosomal dysfunction. P53 activation can also mediate tumor cytotoxicity mediated by reduction in levels of certain ribosome proteins. Decreased levels of RPS9 lead to growth inhibition in osteosarcoma cell lines. It has been shown that depletion of RPS9 provokes a rapid loss of the nucleolar protein pool, impaired production of mature 18 S ribosomal RNA and activation of the p53 tumor suppressor pathway. The combination of a defective ribosome biogenesis pathway and p53 activation results in unexpectedly strong anti-proliferative responses in human tumor cell lines [64]. While decreased expression of some ribosomal proteins can activate p53 as part of a cellular stress response, it is also evident that some other ribosomal proteins have direct and more specific roles in *p53* mRNA translation. Haploinsufficiency of a subset of r-proteins (L35, S15a, S8, L36, L7, S7, L13, S29, L23a) in zebrafish is linked to the development of malignant peripheral nerve sheath tumors. Interestingly, these nerve sheath tumors fail to produce p53 protein, despite presence of *p53* mRNA [65]. Moreover, RPL26 plays a role in enhancing *p53* mRNA translation during the DNA damage response by direct interactions with both MDM2 protein and *p53* mRNA [66].Hence, ribosomal proteins have more dynamic roles in regulating the p53 tumor suppressor pathway than was previously thought and this complex interaction appears to be the most significant extraribosomal function of the ribosomal proteins, through which they exert an effect on the cell cycle and apoptosis. It could explain not only some features of ribosomopathies (particularly DBA and 5q- syndrome) but may also have a significant role in carcinogenesis (as elicited in the RPS9 silencing study).

3. Non p53 mediated extraribosomal functions of ribosomal proteins in cancer:There is some evidence showing that other extraribosomal function of ribosomal proteins may also be directly involved in tumorigenesis. RPs can directly regulate the affect of oncogene and tumor suppressors on DNA replication, transcription and translation. The recombination of human trk proto-oncogene with RPL7a activates its oncogenic function [67]. RPS29, whose mRNAs is much higher in quiescent cells than that in growth phase of endothelial cells, has tumor suppressor activity for ras transformed NIH3T3 cells [68]. On the other hand, the overexpression of RPS3a gene has been shown to promote tumorigenesis in nude mice works via suppression of apoptosis by inducing synthesis of anti-apoptosis proteins [69]. These studies indicate that extraribosomal functions of distinct ribosomal proteins may also regulate tumorigenesis.

## Conclusions

Ribosomal protein dysfunction has been shown to be associated with the haematological manifestations of Diamond Blackfan Anemia, 5q- syndrome and facial features of Treacher-Collins syndrome (and possibly that of DBA as the facial features of the two conditions are very similar). Interestingly, all these features have been shown to be caused by a deranged RP-MDM2-p53 pathway. Development of therapeutic targets against this pathway could possibly reverse the hematological manifestations. In Cartilage Hair Hypoplasia, Shwachman Diamond syndrome and Dyskeratosis Congenita, although there are significant ribosomal dysfunctions found, there still remains a small doubt whether they are causally associated with the conditions, particularly given the fact that the genes involved have other functions, which could also explain the clinical manifestations.

Ribosomal protein disorders in malignancies seem to be more complex. Perturbation in extraribosomal functions of ribosomal proteins seems to be directly involved in tumorigenesis and deranged ribosomal function seems to be the consequence or an associated feature. While some ribosomal proteins have been shown to exert a direct effect on proto-oncogenes and tumorigenesis, others interact directly or indirectly with the p53 tumor suppressor pathway. Further mechanistic studies will reveal distinct sets of ribosomal proteins that are deranged in specific malignancies and will potentially uncover newer therapeutic targets.

## Competing interests

We have no competing interests.

## Authors’ contributions

NS wrote the article, RK, TB, SB, YY performed literature search and contributed to the text, AV wrote the article. All authors have read and approved the final manuscript.
